# Early Growth Inhibition Is Followed by Increased Metastatic Disease with Vitamin D (Calcitriol) Treatment in the TRAMP Model of Prostate Cancer

**DOI:** 10.1371/journal.pone.0089555

**Published:** 2014-02-26

**Authors:** Adebusola Alagbala Ajibade, Jason S. Kirk, Ellen Karasik, Bryan Gillard, Michael T. Moser, Candace S. Johnson, Donald L. Trump, Barbara A. Foster

**Affiliations:** 1 Department of Pharmacology, Roswell Park Cancer Institute, Buffalo, New York, United States of America; 2 Department of Medicine, Roswell Park Cancer Institute, Buffalo, New York, United States of America; University of Central Florida, United States of America

## Abstract

The active metabolite of vitamin D_3_, 1,25-dihydroxyvitamin D_3_ (calcitriol) has antiproliferative effects in non-aggressive prostate cancer, however, its effects in more aggressive model systems are still unclear. In these studies, effects of calcitriol and a less-calcemic vitamin D analog, QW-1624F_2_-2 (QW), were tested *in vivo*, using the aggressive autochthonous transgenic adenocarcinoma of mouse prostate (TRAMP) model. To study prevention of androgen-stimulated prostate cancer, vehicle, calcitriol (20 µg/kg), or QW (50 µg/kg) were administered to 4 week-old TRAMP mice intraperitoneal (i.p.) 3×/week on a MWF schedule for 14 weeks. Calcitriol and QW slowed progression of prostate cancer as indicated by reduced urogenital tract (p = 0.0022, calcitriol; p = 0.0009, QW) and prostate weights (p = 0.0178, calcitriol; p = 0.0086, QW). However, only calcitriol increased expression of the pro-differentiation marker, cadherin 1 (p = 0.0086), and reduced tumor proliferation (p = 0.0467). By contrast, neither vitamin D analog had any effect on castration resistant prostate cancer in mice treated pre- or post-castration. Interestingly, although vitamin D showed inhibitory activity against primary tumors in hormone-intact mice, distant organ metastases seemed to be enhanced following treatment (p = 0.0823). Therefore, TRAMP mice were treated long-term with calcitriol to further examine effects on metastasis. Calcitriol significantly increased the number of distant organ metastases when mice were treated from 4 weeks-of-age until development of palpable tumors (20–25 weeks-of-age)(p = 0.0003). Overall, data suggest that early intervention with vitamin D in TRAMP slowed androgen-stimulated tumor progression, but prolonged treatment resulted in development of a resistant and more aggressive disease associated with increased distant organ metastasis.

## Introduction

Prostate cancer (PCa) is one of the leading causes of cancer and cancer-related mortality in American men [1]. Early stage, organ-confined, PCa may be managed by surgery, radiotherapy or watchful waiting, while men with locally advanced or metastatic PCa are treated primarily by androgen deprivation therapy [2]. However, many patients eventually develop castration resistant PCa, for which current treatment options are only palliative. The prevalence, long latency, morbidity and mortality associated with PCa have generated significant interest in developing agents for chemoprevention of the disease [3].

Epidemiological associations between vitamin D and PCa suggest that vitamin D may be an important regulator of PCa growth and differentiation [4]. However, studies linking serum levels of the vitamin D and PCa incidence and mortality are not strongly correlated (reviewed in [5]). In contrast, preclinical studies demonstrate that vitamin D has potent anticancer effects in PCa cells *in vitro*, including inhibition of cell proliferation [6] and invasiveness [7], induction of cell cycle arrest [8], stimulation of apoptosis [9], and promotion of differentiation [10]. Furthermore, numerous *in vivo* studies utilizing mouse xenograft systems demonstrate that vitamin D compounds suppress tumor growth and metastasis [6], [11], [12]. Additionally, use of genetically engineered mouse models that spontaneously develop cancer, demonstrate that vitamin D can prevent progression of pre-cancerous PCa lesions [13] and suppress tumor growth in castration resistant PCa [14].

Clinical trials performed in PCa patients have been mostly disappointing [15], [16], [17], [18], although there have been some positive results [19], [20], and gains have been made in the understanding of vitamin D pharmacokinetics and pharmacodynamics. One reason for poor clinical performance of vitamin D is its major dose-limiting toxicity, hypercalcemia. Although hypercalcemia is easily managed, it may be preventing the optimal utilization of calcitriol. Therefore, less calcemic vitamin D analogs have been developed with the intention of retaining the potent anticancer effects of calcitriol. QW-1624F_2_-2 (QW) is a fluorinated hybrid analog of calcitriol that is 100 times less-calcemic, highly antiproliferative, and elicits similar downstream effects to calcitriol [21], [22]. In addition, administration of high doses of QW (3 µg) inhibit 7,12-dimethylbenz[a]anthracene-initiated and TPA-promoted skin carcinogenesis in mice without hypercalcemic side effects [23].

The transgenic adenocarcinoma of mouse prostate (TRAMP) model is an aggressive, autochthonous model of PCa [24]. By 10 weeks-of-age, TRAMP mice develop low grade, non-invasive prostatic cancer [25]. As mice continue to age, the tumor progresses from non-invasive to high grade, invasive adenocarcinoma, and subsequently poorly differentiated neuroendocrine tumors by 20-25 weeks-of-age. The progressive nature of the model presents an opportunity to examine the effects of chemopreventive agents on both early and late stages of PCa development, as opposed to only one or the other. Thus far, head-on comparisons of the chemopreventive effects of different vitamin D compounds on androgen-stimulated and castration-resistant PCa progression have yet to be conducted. Since prostate cancer mortality occurs in men with advanced, late-stage cancer, we sought to examine the anti-cancer efficacy of vitamin D compounds using the more aggressive TRAMP model. In these studies, the TRAMP model was used to investigate whether calcitriol or QW can prevent or slow androgen-stimulated and castration-resistant PCa initiation, progression and metastasis.

## Materials and Methods

### Ethics Statement

Experimental uses of laboratory animals were conducted in strict accordance with the National Institutes of Health Guide for the Care and Use of Laboratory Animals and the protocol was approved by the Roswell Park Cancer Institute (RPCI) Animal Care and use Committee (Assurance # A3143-01). RPCI is an AALAC International accredited animal research facility. All surgery was performed using isoflurane for inhalational anesthesia and all efforts were made to minimize animal suffering.

### Animals

Breeding colonies were maintained at the Department of Laboratory and Animal Resource (DLAR) core facility at Roswell Park Cancer Institute (Buffalo, NY). Homozygous male TRAMP mice were bred with wild-type FVB female mice (Taconic, Germantown, NY) to obtain C57BL/6×FVB 50∶50 male TRAMP mice. Mice were genotyped to confirm germ-line transmission of the transgene [24]. All experimental animals were heterozygous for the transgene, and maintained on the Harlan Teklan Diet S2335 that contains 2,980 IU/kg of vitamin D3.

### Vitamin D chemoprevention studies

1α-hydroxymethyl-16-ene-24,24-difluoro-25-hydroxy-26,27-bis-homovitamin D_3 (_QW-1624F_2_-2, QW) was obtained from Dr. Gary Posner (Johns Hopkins University, Baltimore, MD). Calcitriol and QW stocks were dissolved in 100% ethanol, and were freshly resuspended in saline before each injection. Animals were weighed, monitored for toxicity, and palpated to assess tumor burden on a weekly basis. Studies were generally terminated 72 h after the last drug treatment was administered. At the time of euthanasia, final body weights were measured and the urogenital (UG) tract (bladder, seminal vesicle and prostate) was excised and weighed. The dorsal, lateral and ventral prostate lobes were microdissected from the UG tract and weighed to obtain prostate weights. Metastatic incidence was determined based on gross examination of the pelvic lymph nodes, livers, kidneys and lungs, and by immunohistochemical staining of tissue with SV40 T antigen specific antibody. The following tissues were collected in 9-chamber cassettes for histology: dorsal, lateral, ventral and anterior prostates, seminal vesicle, peri-aortic lymph nodes, kidney, intestine and liver. Collection of tissues for histology was prioritized, thus, only leftover tissues from large prostate tumors were snap-frozen in liquid nitrogen and stored at −80°C for Western blot analysis.

**Figure 1:**
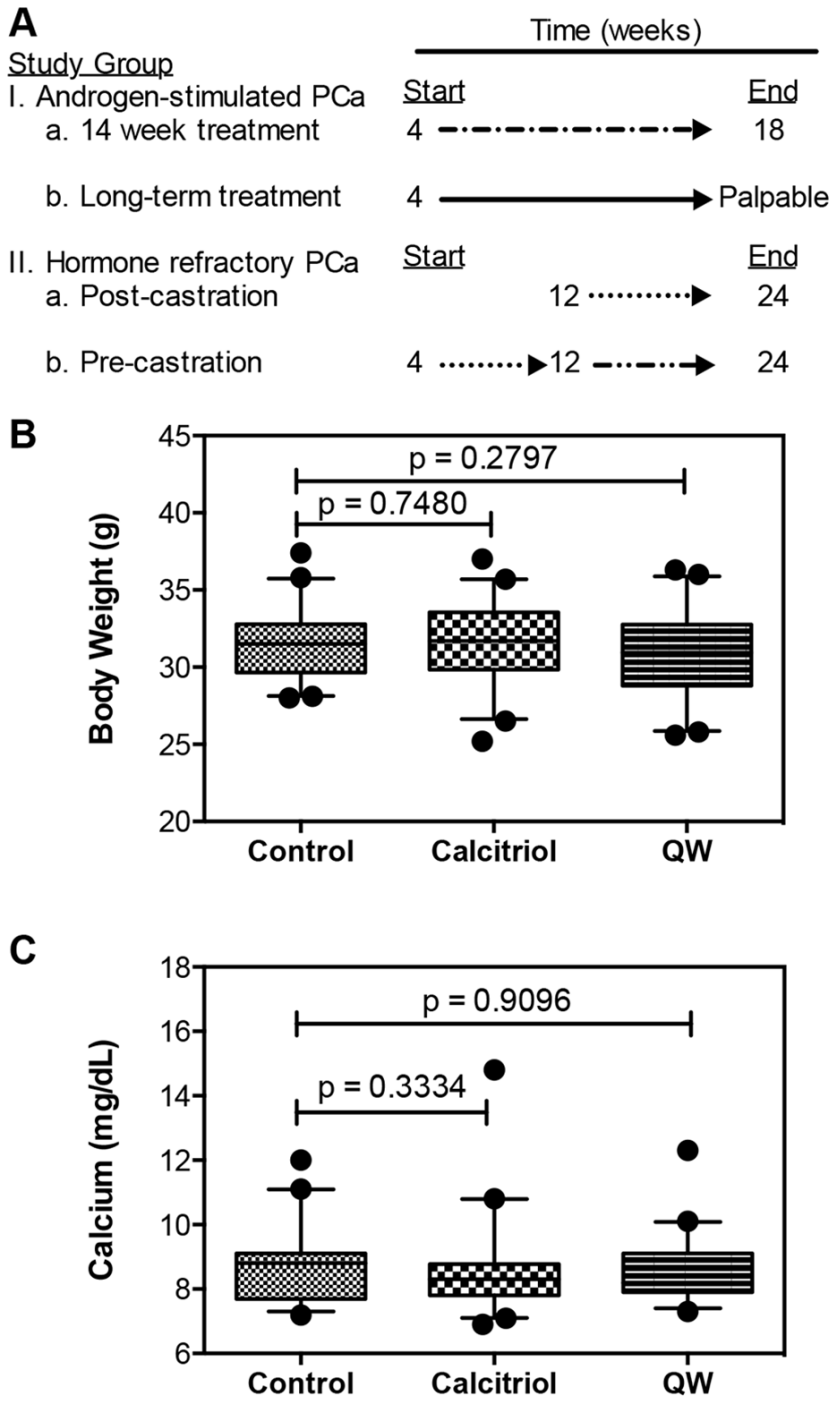
Schematic of prevention studies and safety of vitamin D compounds in TRAMP mice. (**A**) Schematic of the time course of chemoprevention studies. All mice were castrated at 12 weeks in the hormone refractory studies. (**B–C**) Four week-old TRAMP mice were treated with vehicle (control), calcitriol (20 µg/kg) or QW (50 µg/kg) i.p. 3×/week for 14 weeks. (**B**) Final body weights (g) of control (n = 40), calcitriol (n = 41) and QW (n = 42) mice measured 72 h after last treatment. (**C**) Serum calcium (mg/dL) levels for control (n = 39), calcitriol (n = 40) and QW (n = 42) mice measured 72 h after last treatment. All data are represented as box plots with medians, and whiskers representing the 5^th^ and 95^th^ percentiles. • (dots), represent points outside the 5^th^ and 95^th^ percentile. All p-values were generated using the Mann-Whitney test.

To study the effect of calcitriol or QW on progression of androgen-stimulated PCa, three cohorts of 4 week-old TRAMP mice were treated with vehicle (n = 40), calcitriol (n = 41) or QW (n = 42) i.p. 3×/week Monday, Wednesday and Friday (MWF) for 14 weeks (Figure 1A). The study was terminated when mice were 18 weeks old and tissues were harvested as described above. Some samples were unable to be analyzed, or not evaluated based on stated exclusion criteria. A second study was conducted to investigate the effect of *long-term* administration of calcitriol on androgen-stimulated PCa progression. Two cohorts of 4 week-old TRAMP mice were treated *long-term* with vehicle (n = 47) or calcitriol (n = 49) i.p. 3×/week MWF, until palpable tumors developed (Figure 1A). Tumors were harvested when detectable by abdominal palpation.

Two studies were designed to evaluate the effect of calcitriol or QW on prevention of castration resistant PCa in surgically castrated TRAMP mice. In the *post-castration* study, treatment was initiated immediately following castration of TRAMP mice at 12 weeks-of-age. Three cohorts of 12 week-old castrated mice were treated with vehicle (n = 33), calcitriol (n = 31) or QW (n = 30) i.p. 3×/week MWF for 12 weeks (Figure 1A). Mice were euthanized at 24 weeks-of-age and tissue samples were procured as described above. The *pre-castration* study, 4 week-old TRAMP mice were treated with vehicle (n = 29) or calcitriol (n = 34) i.p. 3×/week MWF for 8 weeks. At 12 weeks-of-age, all mice were castrated and treatment was continued for an additional 12 weeks (Figure 1A). Mice were euthanized at 24 weeks-of-age and tissue samples were procured as described above.

### Histopathological analysis

Tissues were harvested, fixed, processed, paraffin-embedded and tissue sections (5 µm) were cut for histological analysis. Hematoxylin and eosin (H & E) staining of tissue sections was performed and tumor grades were assigned by blinded evaluation by A.A.A. and B.A.F. using a standard histopathological grading system developed for the TRAMP model [25]. In this grading system, the normal prostate (grade 1) progressively develops prostatic lesions including low-grade prostatic intraepithelial neoplasia (PIN) (grade 2), carcinoma *in situ* (grade 3), invasive cancer (grades 4 and 5) and poorly differentiated late-stage cancer (grade 6) [25]. The terminologies used to describe prostatic lesions in the current studies were adapted from the aforementioned grading system, where *low-grade* (LG) lesions are equivalent to tumor grades 1 and 2, *intermediate grade* (IG) lesions represent grade 3 and *cancer* is equivalent to grades 4, 5 and 6. The dorsal, lateral and ventral prostate lobes were individually graded and assigned an overall grade and worst grade. The overall grade is determined by assessing the predominant tumor grade present in each prostate lobe. Metastatic incidence was confirmed by examining the lymph nodes, livers, kidneys, and lungs under a light microscope after immunohistochemical staining for SV40 large T antigen. Representative photomicrographs were taken using a KP-D50 color digital camera (Hitachi, Brisbane, CA).

### Immunohistochemistry

Tissue sections were de-paraffinized using xylene and rehydrated with graded alcohols. Antigen retrieval was performed by boiling with 1× Citrate Buffer, pH 6.0 (Zymed Laboratories, San Francisco, CA) for 20 min. Endogenous peroxidase was blocked by immersing slides in 3% H_2_O_2_ in methanol for 15 min. Slides were incubated with the following primary antibodies: SV40 large T antigen (Tag) (1∶400, BD Pharmingen, San Diego, CA), MKI67 (1∶1000, Novocastra Laboratories, Newcastle upon Tyne, UK), cadherin 1 (CDH1) (1∶500, BD Pharmingen), androgen receptor (AR) (1∶200, Upstate, Lake Placid, NY) and synaptophysin (SYP) (1∶400, Zymed). Secondary antibody only was used as negative control for all antibodies. Slides were washed with 1× Tris phosphate buffer, pH 7.8 (8.4 mM Na_2_HPO_4_, 3.5 mM KH_2_PO_4_, 10 mM Tris, 120 mM NaCl) followed by incubation with the following secondary antibodies: HRP-conjugated rabbit anti-mouse (DakoCytomation, Carpinteria, CA), HRP-conjugated swine anti-rabbit (DakoCytomation) and biotinylated goat anti-rabbit (Vector Laboratories, Burlingame, CA). Immunoreactivity was detected using Vectastain ABC reagent (Vector Laboratories) and 3,3′-diaminobenzidine tetrahydrochloride (DAB, Sigma, St. Louis, MO). Tissues were counterstained with hematoxylin, dehydrated with graded alcohols, cleared with xylene and mounted using glass coverslips.

The CDH1 score of the ventral lobe was calculated by counting the number of CDH1 positive glands per 15 glands in a 10× field. The proliferative index of the ventral lobe was determined by counting the number of MKI67 positive cells in three fields (40× magnification) that were representative of areas with the worst tumor grade, divided by the total number of nuclei in each field.

### Western blot analysis

Immunoblotting was performed as previously described [22]. Briefly, snap-frozen tissues were homogenized with triton-X/SDS lysis buffer containing 1× Protease Inhibitor Cocktail (BD PharMingen) and phosphatase inhibitors. Samples were resolved by SDS-PAGE and transferred to PVDF membranes overnight at 4°C by electrophoresis. Membranes were blocked at room temperature for at least 1 h using 5% (w/v) milk in 1× TBST. Following overnight incubation with primary antibodies for SV40 large T antigen (1∶250, BD Pharmingen) and actin (CP01, Calbiochem, San Diego, CA), membranes were washed with TBST, incubated with HRP-conjugated secondary antibodies for a minimum of 1 h and protein expression was detected by chemiluminescence.

### Serum analysis

Blood was obtained by terminal cardiac puncture at the time of necropsy and serum was collected from blood samples by centrifugation. Calcium levels were measured at the Roswell Park Cancer Institute Clinical Laboratories.

### Statistical analysis

All graphs and statistical analysis were generated using GraphPad Prism version 5.0d for Mac OS X, GraphPad Software, San Diego California USA, www.graphpad.com. The nonparametric Mann-Whitney test was performed to compare between quantitative outcomes, while categorical analysis was performed by developing contingency tables and using Chi-Square or Fisher's Exact tests. Quantitative results are represented as box plots that depict the 5^th^, 25^th^, 50^th^, 75^th^ and 95^th^ percentiles of data sets in each treatment group, and categorical results are represented as percentages of each treatment group.

## Results

### Vitamin D compounds are non-toxic and do not interfere with SV40 Large T antigen expression

**Figure 2:**
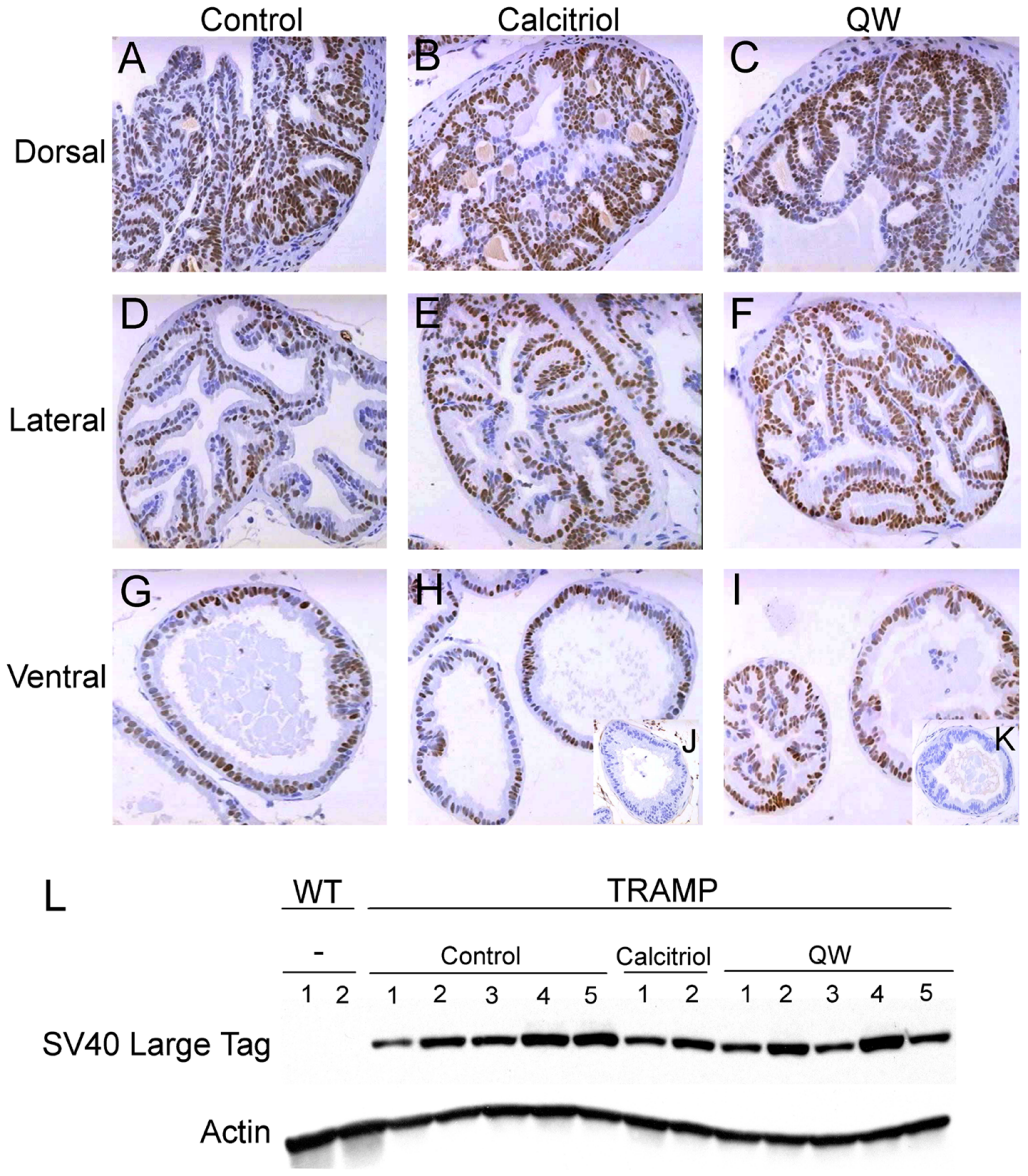
Expression of SV40 large T antigen (Tag) in prostate tissues. TRAMP mice were treated with vehicle (control), calcitriol (20 µg/kg) or QW (50 µg/kg) i.p. 3×/week for 14 weeks. Immunohistochemical analysis of Tag expression in the dorsal (**A–C**), lateral (**D–F**), and ventral (**G–I**) lobes of 18 week-old mice. (**J**) SV40 Tag staining of the ventral lobe of age-matched non-transgenic mice. (**K**) No antibody staining negative control. Photomicrographs were taken at 20× magnification. (**L**) Western blot analysis of Tag expression in poorly differentiated prostate tumor samples of control, calcitriol- and QW-treated mice. Actin was used as loading control. WT, Wild-type and age-matched non-transgenic prostate tissues. The sample number used for immunoblotting varies (control  = 5, calcitriol  = 2, QW = 5) because priority was given to collecting tissues for histology. Only leftover tissues from large prostate tumors were available for immunoblotting.

The effects of vitamin D compounds on progression of androgen-stimulated PCa were evaluated in mice at sexual maturity, which begins at approximately 4 weeks-of-age in TRAMP. Three cohorts of 4 week-old TRAMP mice were treated with calcitriol (20 µg/kg), QW (50 µg/kg) or vehicle for 14 weeks on a MWF schedule (Figure 1A). Mice treated with vitamin D compounds did not experience any weight loss following therapy (Figure 1B). Final body weights were 31.5±2.1 g for vehicle, 31.5±2.5 g for the calcitriol, and 30.7±2.7 g for QW treated mice. Furthermore, calcium levels were unchanged after 14 weeks of treatment with vehicle (8.6±1.0 mg/dL), calcitriol (8.5±1.4 mg/dL, p = 0.3334), or QW (8.5±0.9 mg/dL, p = 0.9096), suggesting sustained hypercalcemia is not a problem (Figure 1C). Calcium levels were elevated in a few tumor-bearing mice (vehicle control, n = 2/39; calcitriol, n = 1/40; and QW, n = 1/42), but this is likely related to the tumor phenotype and independent of treatment. Because prostate-specific expression of the SV40 early genes (T and t antigens; Tag) is necessary for tumor progression in TRAMP [24], it was important to determine whether therapeutic agents alter transgene expression. Similar to the control group (Figures 2A, D, G), Tag was expressed in the dorsal (Figures 2B–C), lateral (Figures 2E–F) and ventral (Figures 2H–I) prostate lobes following treatment with calcitriol or QW respectively. Western blot analysis further confirmed that vitamin D compounds did not interfere with Tag expression (Figure 2L).

### Effect of vitamin D compounds on tumor incidence and progression

The ability of calcitriol or QW to inhibit or delay tumor formation in TRAMP was analyzed after treatment of androgen-stimulated and castration resistant PCa (Figure 1A). In androgen-stimulated mice treated with vehicle, calcitriol, or QW, from 4 weeks-of-age until 18 weeks-of-age, tumor incidence was evident in 35.0% of vehicle control mice (n = 14/40), 26.8% of calcitriol treated mice (n = 11/41) and 35.6% of QW treated mice (n = 15/42) (Figure S1). Although there was an 8.2% decrease in tumor incidence with calcitriol treatment, this decrease was not significant (p = 0.6337, Chi-Square test).

Following castration at 12 weeks-of-age, TRAMP mice develop castration resistant tumors by 24 weeks-of-age [25], [26]. To determine if vitamin D compounds can slow or prevent PCa recurrence, three cohorts of castrate TRAMP mice were treated with vehicle, calcitriol (20 µg/kg), or QW (50 µg/kg) for 12 weeks *post-castration* (Figure 1A). Approximately 64% (21/33) of vehicle control mice developed castration resistant tumors, compared to 68% (21/31) and 57% (17/30) of mice in the calcitriol and QW groups respectively (Figure S1). However, these changes were not significant (p = 0.6648, Chi-Square test). We also investigated whether intervention with vitamin D treatment *pre-castration* could alter castration recurrence. Two cohorts of 4 week-old TRAMP mice were treated with either vehicle or calcitriol (20 µg/kg) starting at 4 weeks-of-age. At 12 weeks-of-age mice were castrated and therapy was continued until mice were 24 weeks-of-age (Figure 1A). Approximately 62% (18/29) of vehicle treated mice and 74% (25/34) of calcitriol-treated mice developed castration resistant disease (Figure S1), but no significant difference was observed (p = 0.4184, Fisher's Exact test). Overall, these results indicate that vitamin D compounds were unable to reduce recurrence of castration resistant PCa in TRAMP.

### Effect of vitamin D compounds on tumor grade and disease burden

**Figure 3:**
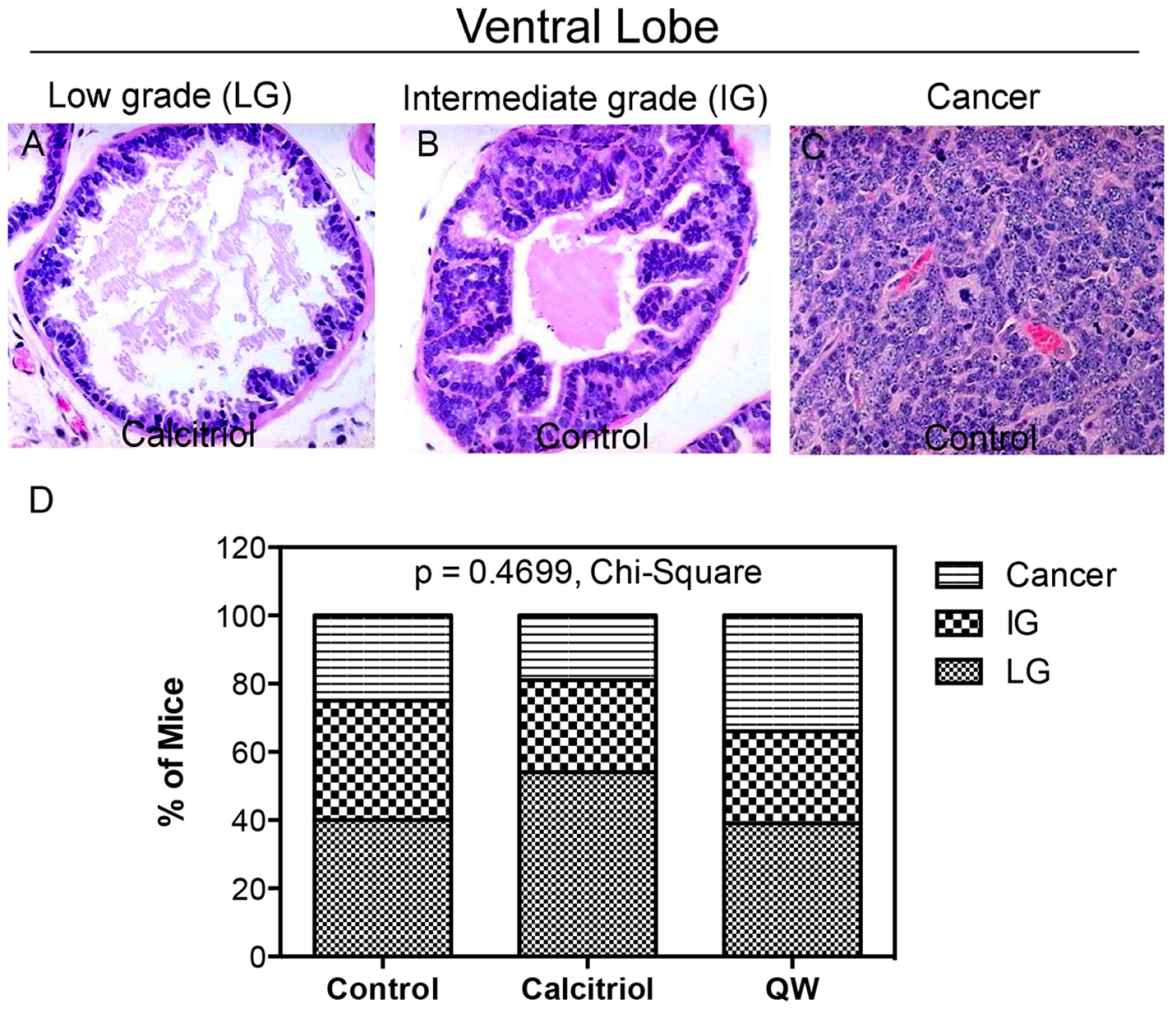
Effect of vitamin D compounds on tumor grade in the ventral lobe of TRAMP mice. H & E staining was performed on tissue sections from TRAMP mice treated with vehicle (control), calcitriol (20 µg/kg) or QW (50 µg/kg) i.p. 3×/week for 14 weeks. Prostate tissues were graded based on a histopathological grading system discussed in the *Materials and Methods* section. Representative photomicrographs (20× magnification) of H & E staining of ventral lobes depicting (**A**) Low grade (LG) prostatic lesion from the calcitriol group, (**B**) intermediate grade (IG) lesion, and (**C**) late-stage cancer in the control group. (**D**) Incidence (%) of LG lesions, IG lesions, and cancer in the ventral lobes of control (n = 40), calcitriol (n = 41) and QW (n = 41) groups. A Chi-Square test was performed to determine if there was any association between treatment and disease stage.

To further assess the effects of vitamin D compounds on androgen-stimulated PCa progression in mice following 14 weeks of therapy, prostatic disease was scored by standard histological evaluation (see *Materials and Methods*). Overall tumor grades were assigned following blinded examination of H & E-stained slides. Prior reports demonstrate that the ventral lobe is most sensitive to therapeutic intervention [27], [28], therefore overall tumor grades of the ventral prostate were compared between treatment groups (Figure 3). Low grade (LG) lesions were non-invasive and were characterized by increased epithelial stratification with intact glandular architecture (Figure 3A). Intermediate grade (IG) lesions were also non-invasive, but displayed prominent nucleoli, epithelial tufting, micropapillary structures, cribriform architecture, and an intact glandular architecture (Figure 3B). Lastly, late-stage cancers prominently consisted of sheets of anaplastic cells with loss of glandular architecture (Figure 3C). The incidence of LG lesions was 40% in vehicle treated, 54% in the calcitriol treated, and 39% in QW treated mice (Figure 3D). In contrast, IG lesions and late-stage cancer developed more frequently in the control (60%) and QW (61%) groups compared to calcitriol-treated mice (46%) (Figure 3D). Furthermore, the incidence of cancerous lesions was 25% in the control, 34% in the QW group, and 20% in the calcitriol group (Figure 3D), however these changes were not significant (p = 0.4699, Chi-Square test). Intervention also had no effect on tumor grade in dorsal and lateral lobes of the prostate (data not shown).

**Figure 4:**
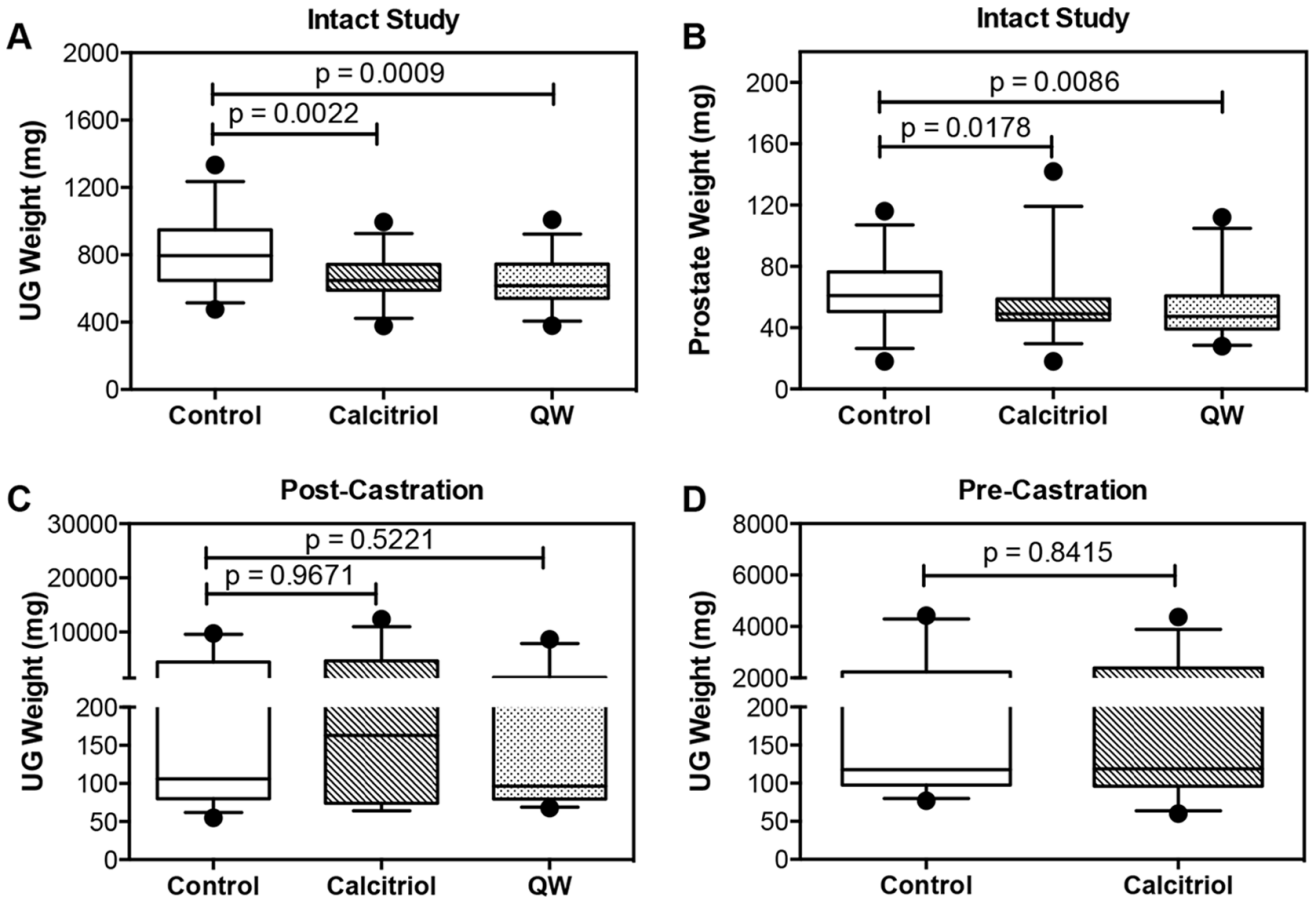
Effect of vitamin D compounds on disease progression in TRAMP mice. (**A–B**) Mice were treated with vehicle (control), calcitriol (20 µg/kg) or QW (50 µg/kg) i.p. 3×/week for 14 weeks. Only samples with overall non-invasive low grade (LG) and intermediate grade (IG) prostatic lesions in the dorsal, lateral and ventral lobes were analyzed, while all cancers were excluded from analyses. (**A**) Urogenital (UG) weights (mg) of control (n = 30), calcitriol (n = 32) and QW (n = 28) mice. (**B**) Prostate weights (mg) of control (n = 29), calcitriol (n = 32) and QW (n = 28) groups. (**C**) UG weights (mg) of 24 week-old castrate TRAMP mice treated with vehicle control (n = 33), calcitriol (n = 30) or QW (n = 30) *post-castration*. (**D**) UG weight (mg) of 24 week-old castrate TRAMP mice treated with vehicle control (n = 29) or calcitriol (n = 34) *pre-castration*. All data are represented as box plots with medians, and whiskers representing the 5^th^ and 95^th^ percentiles. • (dots), represent points outside the 5^th^ and 95^th^ percentile. All p-values were generated using the Mann-Whitney test.

Disease burden in TRAMP can be directly linked to prostate and urogenital tract (UG) weight [25]. The effect of vitamin D compounds on disease burden was initially examined in all samples including LG, IG, and late-stage cancers. No significant differences were observed, likely resulting from the development of bulky tumors in late-stage cancers. Once bulky tumors have developed, the weights from these tumors may mask any effect vitamin D has on LG and IG lesions. Therefore, all subsequent analyses performed for androgen-stimulated PCa were done on the non-invasive LG and IG prostatic lesions. Calcitriol (p = 0.0022) and QW (p = 0.0009) significantly decreased UG weight when compared to vehicle in androgen-stimulated disease (Figure 4A). In addition, calcitriol (p = 0.0178) and QW (0.0086) both reduced prostate weight (Figure 4B). However, in castration resistant TRAMP tumors histologically comparable to late-stage cancer, vitamin D compounds did not statistically alter disease burden when treated post-castration (Figure 4C) or pre-castration (Figure 4D). Overall, results suggest that vitamin D compounds were effective in slowing growth of early stage androgen-stimulated PCa, but ineffective in late stage castration resistant TRAMP disease.

### Calcitriol promotes differentiation and inhibits proliferation in androgen-stimulated TRAMP mice

**Figure 5:**
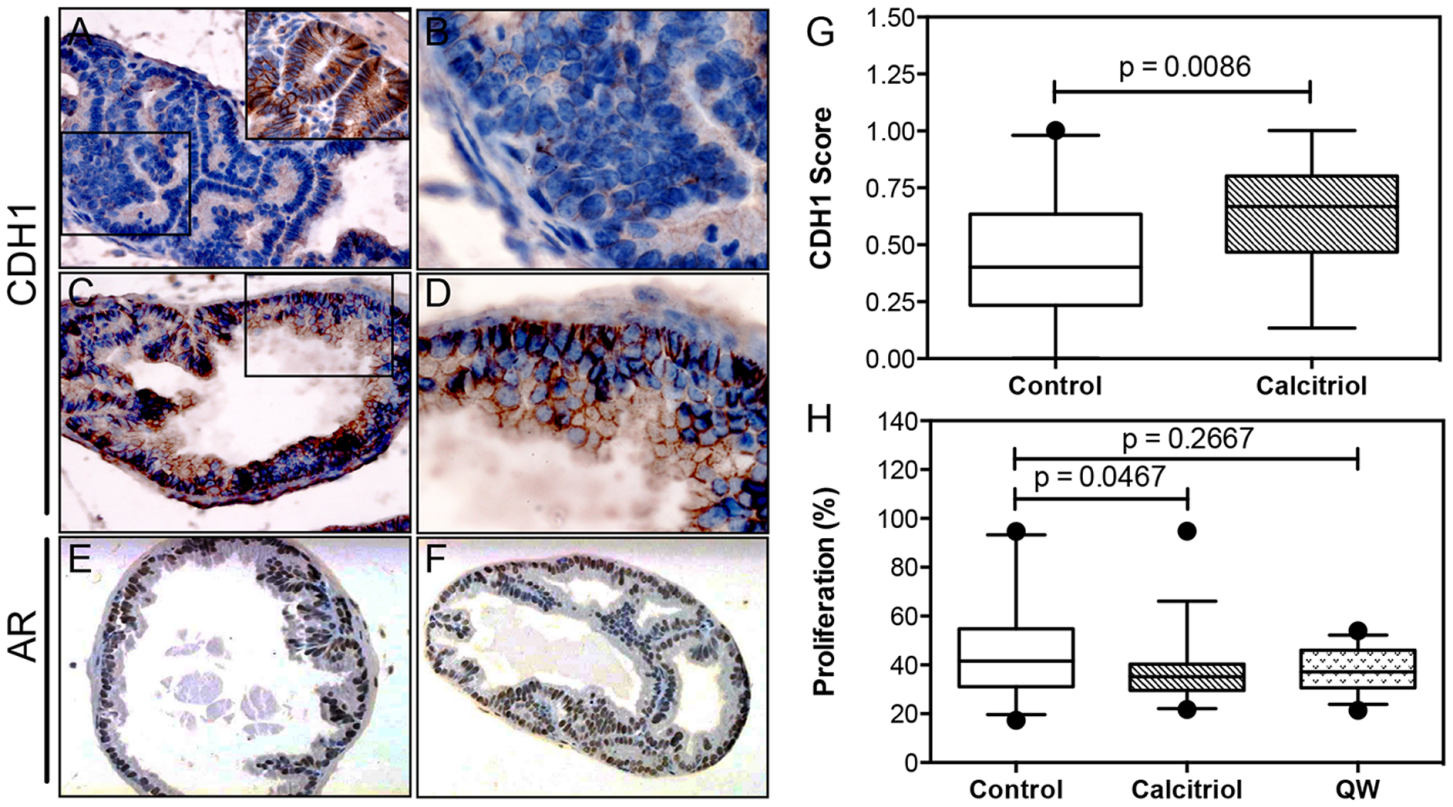
Effect of vitamin D compounds on differentiation and proliferation in TRAMP mice. Immunohistochemistry was performed with anti-CDH1, anti-AR and anti-MKI67 antibodies on tissue sections from control, calcitriol and QW treatment groups. (**A–D, G**) CDH1 expression in non-invasive low grade (LG) and intermediate grade (IG) ventral prostate tissues. Representative photomicrographs of CDH1 staining in control (**A–B**) and calcitriol (**C–D**) groups. Inset in (**A**) represents positive CDH1 staining of normal colon epithelial cells. Photomicrographs were taken at 40× magnification (**A,C**) and boxed areas represent the 100× magnification (**B,D**). Representative photomicrographs (40× magnification) of AR expression in non-invasive prostatic lesions from ventral lobes of (**E**) control and (**F**) calcitriol group. (**G**) CDH1 score of non-invasive ventral lobe lesions from control (n = 25) and calcitriol (n = 27) groups was calculated by quantifying number of E-Cadherin positive glands as described in *Materials and Methods* section. (**H**) Proliferative index of LG and IG ventral lobe lesions of control (n = 30), calcitriol (n = 33) and QW (n = 27) groups was determined by quantifying MKI67 staining as described in *Materials and Methods* section. Only LG and IG prostatic lesions were analyzed, while late-stage cancers were excluded for both CDH1 scoring and MKI67 staining. All data are represented as box plots with medians, and whiskers representing the 5^th^ and 95^th^ percentiles. • (dots), represent points outside the 5^th^ and 95^th^ percentile. All p-values were generated using the Mann-Whitney test.

Based on observations that calcitriol inhibited UG and prostate weight in the ventral lobe (Figure 4), further analyses were performed in non-invasive LG and IG lesions. Loss of cadherin 1 (CDH1) expression is associated with PCa progression and decreased differentiation in TRAMP [25], [29]. CDH1 expression is predominantly located to the plasma membrane. However, when cancer cells lose differentiation status, regulation of CDH1 can be disrupted resulting in cytosolic and nuclear expression patterns [30]. Furthermore, increases in expression of CDH1 correlate with induction of differentiation induced by vitamin D analogs in PCa cell lines [31]. Therefore, expression of CDH1 was assessed to determine the effect of calcitriol on differentiation. In contrast to control mice (Figures 5A–B), CDH1 was strongly expressed in the ventral lobes of calcitriol treated mice (Figures 5C–D). While some cytoplasmic staining of CDH1 was observed in both treatment groups, the majority of CDH1 staining remained membrane bound. Subsequent quantification confirmed that calcitriol significantly increased the number of CDH1 positive glands compared to vehicle (Figure 5G)(p = 0.0086). In addition to CDH1, androgen receptor (AR) expression was also examined in the ventral prostates of vehicle and calcitriol treated mice (Figures 5E and 5F). Although no difference in AR expression was observed, it is important to note that AR expression was maintained. Finally, there is a stochastic emergence of neuroendocrine cancers in the TRAMP model that is marked by increased expression of synaptophysin during tumor progression [25]. Examination of synaptophysin expression indicated that calcitriol and QW treatment did not alter incidence of the neuroendocrine phenotype (data not shown).

In addition to examining the differentiation status of LG and IG ventral lobes, immunohistochemical staining was performed with a MKI67 antibody to examine the effect of calcitriol and QW on proliferation of the ventral lobe. Compared to vehicle, calcitriol significantly decreased proliferation in the ventral lobe (p = 0.0467), while QW was ineffective (p = 0.2667) (Figure 5H). Analysis of apoptosis demonstrated that the overall expression of pro-apoptotic caspase 3 was very low (data not shown). The low expression of caspase 3 made it difficult to accurately quantify, and suggested that apoptosis was not prevalent in calcitriol treated prostate glands.

### Effect of vitamin D compounds on distant organ metastasis

In addition to examining progression of primary prostatic tumors, appraisal of metastatic disease was also performed. Metastatic disease was initially investigated by gross examination of local lymph nodes and distant organs, including the lung, liver, and kidney. Subsequently, Tag expression was assessed in all lymph nodes, livers, and kidneys by immunohistochemical analysis. Lungs that contained a metastatic lesion upon gross examination where also examined with Tag staining. In TRAMP, Tag expression is controlled by the androgen regulated minimal rat probasin promoter (−426/+28) and expressed specifically in cells originating from the prostate [24], making it a good marker of metastatic disease. The effect of vitamin D compounds on metastasis was performed only in samples from the androgen-stimulated study.

**Figure 6:**
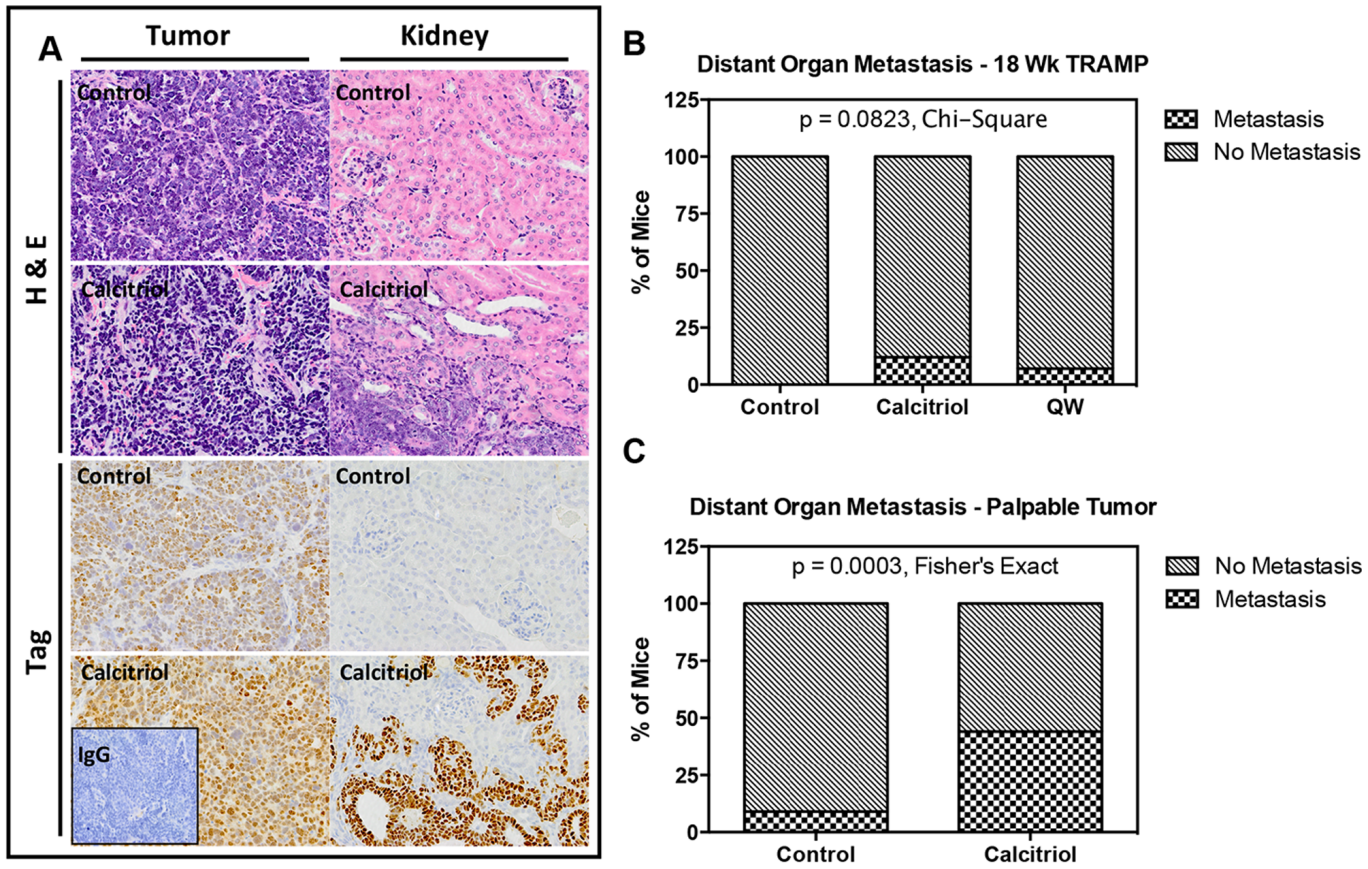
Effect of vitamin D compounds on distant organ metastasis in TRAMP mice. Immunohistochemistry was performed with anti-SV40 large Tag antibody on tissue sections from control, calcitriol, and QW treatment groups. (**A**) Representative H&E and SV40 large T antigen stained prostate tumor and kidney tissue sections from control and calcitriol treated mice. Tag positivity is shown as a brown stain against the background. All photomicrographs are taken at 40× magnification. (**B**) The percentage of mice with distant organ metastases identified by Tag staining in control (0/40), calcitriol (5/41), and QW (3/42) treated TRAMP mice at 18 weeks-of-age. (**C**) The percentage of mice with distant organ metastases identified by Tag staining in control (4/43) and calcitriol (20/45) treated TRAMP mice when treated until development of late stage palpable tumors (20–25 weeks-of-age). Chi-Square tests and Fisher's exact tests were performed to determine if there were any associations between treatment group and development of metastasis.

No difference in lymph node metastasis was detected between vehicle, calcitriol, or QW treated TRAMP mice after 14 weeks of treatment (data not shown). However, analysis revealed that there was a small increase in distant organ metastasis following treatment with vitamin D compounds. Representative images of distant organ metastasis can be observed in H&E and Tag stained histology from primary tumors and matched kidneys from vehicle and calcitriol treated mice (Figure 6A). Kidneys from calcitriol treated mice contained infiltrating Tag positive prostate cancer cells, whereas kidneys from vehicle control mice did not. Overall, there were no distant organ metastases identified in control mice (0/40), but five metastases identified in calcitriol treated mice (5/41), and three metastases identified in QW treated mice (3/42) (Figure 6B). Although these numbers suggest a trend towards increased metastasis, these changes were not significant (p = 0.0823, Chi-Square test).

To further analyze the effect of calcitriol on metastasis two cohorts of mice (vehicle and calcitriol treated) were treated long-term, from 4 weeks-of-age until development of late-stage palpable tumors (20–25 weeks-of-age) (Figure 1A). Results indicate that long-term treatment of TRAMP mice with calcitriol increased distant organ metastasis (20/45, p = 0.003, Fisher's exact test) compared to vehicle control (4/43) (Figure 6C). Furthermore, metastases did not preferentially occur at any of the organ sites we examined (Table 1). Finally, although there was a significant increase in metastasis, no effect on overall survival was observed (data not shown).

**Table 1:** The number of distant organ metastases observed per study and treatment group.

Calcitriol Study	Group	Liver Mets	Kidney Mets	Lung Mets	% Mice with Mets^1^
14 Week Treatment	Control	0/40	0/40	0/40	0.0% (0/40)
	Calcitriol	1/41	4/41	1/41	12.2% (5/41)
	QW	2/42	2/42	2/42	7.1% (3/42)
Long Term Treatment	Control	2/43	1/43	2/43	9.3% (4/43)
	Calcitriol	5/45	18/45	9/45	44.4% (20/45)

1 Number of mice with distant organ metastases per total number of mice per treatment group can be found in the parentheses. Numbers in this category may not match up with previous columns because one mouse can have a kidney, liver, and lung metastases at the same time.

## Discussion

We report here that vitamin D compounds (calcitriol and QW) slowed androgen-stimulated PCa growth when administered at the earliest stages of disease progression in TRAMP mice. These results are consistent with a previous report that calcitriol prevents progression of pre-cancerous PIN lesions to cancerous lesions in the anterior lobes of *Nkx3.1;Pten* mutant mice, a less-aggressive model of PCa [13]. However, prolonged treatment of TRAMP mice with calcitriol resulted in increased distant organ metastases, which is contrary to the chemopreventive effects observed when hormone-intact mice were treated with vitamin D from 4 to 18 weeks-of-age. Therefore, although early intervention with vitamin D slowed the growth of non-aggressive TRAMP disease, long-term treatment resulted in development of more aggressive disease. The long-term effect of calcitriol on metastasis in the TRAMP model is also in direct contrast to many previously published studies in regards to the effect of vitamin D compounds on cancer metastasis [32], [33], [34], [35]. Many variables may account for the observed differences, including the treatment regimen used and the aggressiveness of the model system. In order to fully understand the relevance of these findings, more work identifying the mechanism driving the metastatic phenotype is needed. In addition to testing the effects of vitamin D on androgen-stimulated PCa, we also found castration resistant PCa to be unresponsive to intervention, which may be due to the already aggressive nature of the castration resistant phenotype.

UG and prostate weights increase with age and tumor progression in TRAMP mice [25], thus, the reduction of UG and prostate weights by calcitriol and QW at 18 weeks-of-age indicates that these agents slowed early PCa progression. Previous studies indicate that the chemopreventive efficacy of flutamide [28] and toremifene [27] in TRAMP mice are most striking in the ventral lobes. Consistent with these observations, calcitriol was more effective in the ventral prostate. Specifically, calcitriol reduced proliferation, and increased differentiation in non-invasive ventral prostate lesions.

Sustained hypercalcemia was observed in a few tumor-bearing TRAMP mice (≤5.1%) irrespective of treatment group. Cancer-associated hypercalcemia correlates with increased parathyroid hormone-like peptide (PTHLH) levels [36] and PTHLH levels are elevated during PCa progression [37]. The minor incidences of hypercalcemia observed in this study appear to be vitamin D-independent and may be linked to increases in PTHLH levels during PCa progression in TRAMP mice.

Androgens are necessary for maintaining functional differentiation in the normal prostate [38] via interaction with the AR [39]. Intriguingly, Leman *et al.* reported that in the presence of endogenous androgens, calcitriol suppresses prostatic growth in adult rats, while calcitriol had no effect on prostate weight when administered to castrate rats [40]. AR expression was detectable in the well-differentiated and non-invasive ventral prostate glands of TRAMP mice, suggesting that androgen-driven prostatic differentiation may potentiate the pro-differentiation actions of calcitriol in the prostate. CDH1 is a Ca^2+^-dependent cell adhesion molecule that mediates epithelial cell-cell interactions [41]. Loss of CDH1 expression is associated with PCa progression and decreased differentiation in human PCa [42] and in the TRAMP model [25]. Coincident with findings that calcitriol slowed tumor growth; a significant increase in CDH1 expression was observed. Consequently, calcitriol slows androgen-stimulated PCa progression by maintaining the prostate in a more differentiated state.

Neither calcitriol nor QW exhibited any chemopreventive effects when administered to castrate TRAMP mice *pre*- or *post-castration*. Castration did not obliterate PCa and resistant tumors were unresponsive to the pro-differentiating and antiproliferative effects of vitamin D compounds. By the time the *post-castration* study was initiated in 12 week-old mice, prostates are expected to be transformed, with large populations of tumor cells having lost differentiation markers. One of the mechanisms implicated in development of castration resistant PCa is deregulation of the AR signaling axis [43], leading to selection of mutant AR that promotes survival and proliferation of cancer cells. Han *et al.* reported that castration of TRAMP mice does in fact spontaneously select for somatic mutations in AR and promotes expression of AR variants [44]. Selection for AR mutations following castration of TRAMP mice may disrupt normal androgen/AR signals controlling prostatic differentiation, thus limiting the effects of calcitriol and QW. Furthermore, a previous study indicates that the combination of calcitriol and testosterone promotes prostatic differentiation in castrate rats, while calcitriol alone increases prostate weight and promotes stromal proliferation [45]. Thus, the poor efficacy exhibited by vitamin D compounds administered to castrate mice may be attributable to the fact that depleted androgen levels limited the pro-differentiation effects of calcitriol. Given that androgens are required to maintain functional differentiation in the prostate [38], and that castrate TRAMP animals are unresponsive to calcitriol, we propose that endogenous androgens are needed to augment the growth inhibiting and pro-differentiating effects of calcitriol.

The effect of another vitamin D analog, EB 1089, did show some effects in a castration resistant PCa model [14]. Although Perez-Stable et al. did not show inhibition of tumor development, they did see a decrease in prostate weights from tumors treated with EB 1089. The Gγ/T-15 transgenic model used by Perez-Stable et al., drives expression of Tag specifically to the basal cell population, whereas TRAMP targets expression to the luminal epithelial cells. The different sites of disease origin may contribute to the differential responsiveness observed between the two model systems. Furthermore, as discussed above, TRAMP mice have to go through androgen ablation therapy to become hormone insensitive, whereas the Gγ/T-15 transgenic model is inherently hormone insensitive since basal cells do not express AR. When TRAMP tumors overcome ablation therapy they may become inherently more resistant to chemoprevention than tumors arising from the Gγ/T-15 transgenic model, which does not undergo selective resistance.

Although calcitriol has been utilized either alone [19], [46] or in combination with other cytotoxic agents [47], [48], [49] for treatment of PCa in men with advanced disease, there is limited clinical information on the direct effects of vitamin D on PCa prevention. Calcitriol may decrease prostate specific antigen (PSA) levels in men with castration resistant PCa [46] and may decrease the rate of rise of PSA in men with early recurrent PCa [19], but dose-limiting hypercalcemia and hypercalciuria develops in patients when daily calcitriol is administered. The combination of intermittent high-dose calcitriol with dexamethasone [48], or with docetaxel [47], in men with advanced castration resistant PCa also reduces PSA levels. Although these findings are interesting, results from the present study suggest that vitamin D compounds may play a role in slowing or preventing progression of earlier stages of PCa. Hence, a more effective clinical strategy may be to target earlier stages of clinical disease.

Histologic precursors of PCa are detectable as early as the third and fourth decades in asymptomatic men [50], [51], indicating that PCa may be latent for decades before attaining clinical relevance. Thus chemopreventive regimens that can effectively prevent or retard histologic precursors to overt and clinically evident PCa are desirable. We present evidence that early intervention with vitamin D compounds slowed PCa progression. However, prolonged treatment with calcitriol may select for a more resistant and aggressive form of PCa, leading to increased metastatic disease burden. It is very important to note that our current study used high doses of the most active vitamin D compound, calcitriol. Although we see selection for a more aggressive phenotype with prolonged treatment, these results may not be relevant for chemoprevention studies examining the benefits of vitamin D supplementation with cholecalciferol in vitamin D deficient populations. Increased understanding of the mechanism driving the anti-neoplastic and metastatic phenotypes observed after early intervention in TRAMP progression may assist in determining the best approach to use in maximizing the benefits of vitamin D compounds in PCa patients.

## Supporting Information

Figure S1 **Effect of vitamin D compounds on tumor incidence in TRAMP mice.** (**A**) Tumor incidence (%) in androgen-stimulated TRAMP mice following treatment with vehicle control (n = 40), calcitriol (n = 41), or QW (n = 42) at 18 weeks-of-age. (**B**) Tumor incidence (%) at 24 weeks-of-age in castrate TRAMP mice following *post-castration* treatment with vehicle control (n = 33), calcitriol (n = 31) or QW (n = 30). (**C**) Tumor incidence (%) at 24 weeks-of-age in castrate TRAMP mice following *pre-castration* treatment with vehicle control (n = 29) or calcitriol (n = 34). Chi-Square tests and Fisher's exact tests were performed to determine if there were any associations between treatment group and tumor incidence.
(DOCX)
