# Supplementary material for: Early Growth Inhibition Is Followed by Increased Metastatic Disease with Vitamin D (Calcitriol) Treatment in the TRAMP Model of Prostate Cancer
Source: PLoS One. 2014 Feb 26;9(2):e89555. doi: 10.1371/journal.pone.0089555 (PMC3935875; doi:10.1371/journal.pone.0089555)
Supplement: Figure S1 — Effect of vitamin D compounds on tumor incidence in TRAMP mice. (A) Tumor incidence (%) in androgen-stimulated TRAMP mice following treatment with vehicle control (n = 40), calcitriol (n = 41), or QW (n = 42) at 18 weeks-of-age. (B) Tumor incidence (%) at 24 weeks-of-age in castrate TRAMP mice following post-castration treatment with vehicle control (n = 33), calcitriol (n = 31) or QW (n = 30). (C) Tumor incidence (%) at 24 weeks-of-age in castrate TRAMP mice following pre-castration treatment with vehicle control (n = 29) or calcitriol (n = 34). Chi-Square tests and Fisher's exact tests were performed to determine if there were any associations between treatment group and tumor incidence. (DOCX) [file pone.0089555.s001.docx]

**
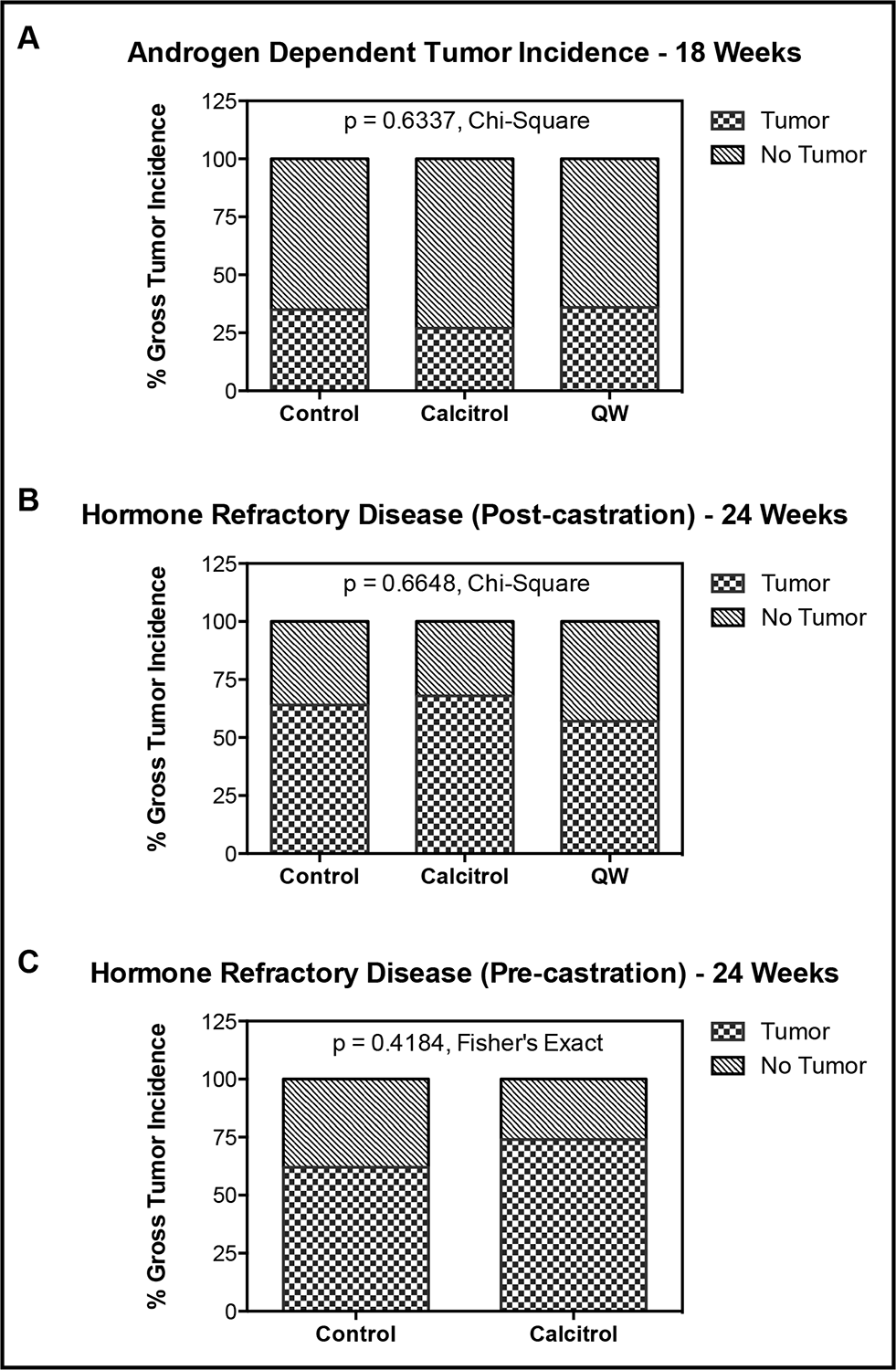
**

**Figure S1. Effect of vitamin D compounds on tumor incidence in androgen-stimulated and castration resistant TRAMP mice. (A)** Tumor incidence (%) in androgen-stimulated TRAMP mice following treatment with vehicle control (n=40), calcitriol (n=41), or QW (n=42) at 18 weeks-of-age. **(B)** Tumor incidence (%) in castrate TRAMP mice following treatment with vehicle control (n = 33), calcitriol (n = 31) or QW (n = 30) *post-castration* at 24 weeks-of-age*.* **(C)** Tumor incidence (%) in castrate TRAMP mice following treatment with vehicle control (n = 29) or calcitriol (n = 34) *pre-castration* at 24 weeks-of-age*.* Chi-Square tests and Fisher’s exact tests were performed to determine if there were any associations between treatment group and tumor incidence.
